# Prenatal Poly I:C exposure affects tryptophan-kynurenine metabolism associated with intestinal microbiome in female juvenile rats

**DOI:** 10.3389/fimmu.2025.1669845

**Published:** 2025-10-01

**Authors:** Lirong Yang, Huiyu Chen, Menglu Zeng, Yanfang Lu, Chen Xu, Zhenju Cao, Fuchun Zhong, Xinyu Yang, Anying Shen, Yueqing Su, Chao Deng, Hua Cao

**Affiliations:** ^1^ School of Public Health, Fujian Medical University, Fuzhou, China; ^2^ Fujian Maternity and Child Health Hospital, Affiliated Hospital of Fujian Medical University, Fuzhou, China; ^3^ College of Clinical Medicine for Obstetrics & Gynecology and Pediatrics, Fujian Medical University, Fuzhou, China; ^4^ School of Medical, Indigenous and Health Sciences, and Molecular Horizons, University of Wollongong, Wollongong, NSW, Australia; ^5^ Fuzhou University Affiliated Provincial Hospital, Fuzhou, China

**Keywords:** maternal immune activation, polyriboinosinic-polyribocytidylic acid, neurodevelopmental disorders, neuroinflammation, tryptophan, gut microbiota

## Abstract

**Background:**

Emerging evidence suggests that disrupted tryptophan (TRP) metabolism may contribute to an increased risk of neurodevelopmental disorders (NDDs) in the context of maternal inflammation, with gut microbiota playing a pivotal role in regulating TRP metabolic pathways.

**Methods:**

Juvenile female rats prenatally exposed to polyriboinosinic-polyribocytidylic acid (Poly I:C) were used to investigate the association between TRP metabolite disturbance and neuropathological/behavioral abnormalities. Behavioral tests assessed anxiety-like behavior, social interaction, and spatial memory. Immunohistochemical and gene expression analyses were performed on the prefrontal cortex (PFC) to evaluate microglial activation and neuroinflammation. TRP-kynurenine (KYN) pathway activity was measured in both PFC and peripheral circulation, along with intestinal TRP metabolism. Gut microbial composition was analyzed through diversity metrics and specific taxa identification.

**Results:**

Prenatal Poly I:C exposure induced anxiety-like behavior, impaired social interaction, and spatial memory deficits in offspring. The PFC showed sustained microglial activation and chronic neuroinflammation. TRP-KYN pathway activation was observed in both central and peripheral systems, accompanied by significant disruptions in intestinal TRP metabolism. Gut microbial analysis revealed reduced diversity and specific alterations in TRP-related taxa (Ruminococcus gauvreauii_group and Candidatus Saccharimonas). These microbial changes correlated with both intestinal TRP metabolic levels and behavioral abnormalities.

**Conclusion:**

Our findings demonstrate that aberrant TRP metabolism associated with gut microbiota dysbiosis contributes to neuroinflammation and behavioral deficits in offspring following prenatal immune activation, highlighting the gut-microbiota-TRP axis as a key mechanism in neurodevelopmental impairments.

## 1 Introduction

Neurodevelopmental disorders (NDDs), including attention-deficit/hyperactivity disorder (ADHD), specific learning disorder (SLD), autism spectrum disorders (ASD), and schizophrenia (SZ), represent a heterogeneous group of neuropsychiatric conditions resulting from disturbance of brain development during early life stages. These disorders typically originate in childhood and result in lifelong impairments in cognition, learning, communication, socialization, or motor skills (1). Maternal immune activation (MIA) refers to the activation of the maternal immune system during pregnancy due to infection, stress, or chemical exposure (2, 3). Epidemiological studies and animal research consistently supported that MIA can affect fetal neurodevelopment, and increase the risk of NDDs in the offspring (3–5). However, the underlying mechanism is still not fully understood.

It is widely known that tryptophan (TRP) is an essential amino acid for the human body and a biosynthetic precursor of various neuroactive compounds (6). There is growing evidence that TRP and its metabolites are crucially affected by acute inflammation and are associated with the disturbance of neurobehavioral development (7–9). Studies have shown that elevated pro-inflammatory cytokines during acute infection upregulate indoleamine 2,3-dioxygenase (Ido), a key rate-limiting cytosolic enzyme in the kynurenine (KYN) pathway that catabolizes TRP. Ido1 has one known homolog, Ido2. Compared to Ido2, Ido1 exhibits significantly higher enzymatic activity and serves a dual protective role: it promotes maternal-fetal immune tolerance through T-cell suppression and helps combat intracellular pathogens via TRP depletion (10, 11). The KYN/TRP ratio serves as an index for assessing the activity of Ido (12). Furthermore, the resulting accumulation of KYN metabolites can act as ligands for the aryl hydrocarbon receptor (AHR), enhancing its activation. This mechanism may exacerbate and sustain a pro-inflammatory state, ultimately impairing neuronal growth and disrupting neuronal circuit structure (10–12). On the other hand, elevated KYN levels may reduce the degradation of endogenous TRP by serotonergic-melatonergic pathways, subsequently decreasing the availability of metabolites that are closely linked to the physiology and pharmacology of neurobehaviors (13). Additionally, since TRP and KYN are transported across the blood-brain barrier (BBB) via specific transporters (14), peripheral circulating KYN can be further metabolized into the neurotoxic Kynurenine 3-monooxygenase (Kmo) branch, producing quinolinic acid (QA), and the neuroprotective Kynurenine aminotransferase II (KatII) branch, generating kynurenic acid (KYNA) (15–18). For example, one study showed a significant increase of L-type amino acid transporter 1 (Lat1) in the brain, which is the key transporter responsible for KYN crossing the BBB (19). Disruption of BBB was observed in several MIA animal models (20). Therefore, it was rationally assumed that MIA-induced long-term pathological changes and neurobehavioral abnormalities in offspring may be attributed to the disruption of TRP metabolism toward the KYN pathway caused by increased proinflammatory cytokines.

Recent discoveries have underscored that the availability of dietary TRP in animals is largely determined by the balance between its catabolism and absorption in the intestine, a process directly or indirectly influenced by the gut microbiota (21, 22). For example, intestinal microbiota plays a major role in the digestion and absorption of nutrients, including TRP-containing proteins (23). Meanwhile, gut microbiota can also directly convert TRP to indole or associated derivatives, such as the common gut Firmicutes, *Clostridium* sp*orogenes*, *Ruminococcus gnavus*, *Lactobacillus*, *Clostridium*, and *Bacteroides* (24–29). Additionally, gut microbial metabolites, such as short-chain fatty acids, may modulate circulating TRP levels by promoting colonic 5-hydroxytryptamine (5-HT) production (30, 31). Furthermore, manipulating the gut microbial composition can influence plasma concentrations of TRP pathway metabolites (30). In turn, variations in TRP metabolism can negatively impact microbial proliferation and microbiota diversity, playing essential roles in the complex interaction between the gut microbiota and hosts (22). For instance, some bacterial TRP metabolites, such as indole-3-aldehyde (IAld), indole-3-acetic acid (IAA), indole-3-propionic acid (IPA), indole-3-acetaldehyde (IAAld), act as ligands for AHR receptors, modulating the endogenous TRP metabolism pathways (32). The key enzyme of Ido1 in the KYN pathway plays a significant negative role in maintaining microbial diversity (33). Over the past decades, numerous clinical studies have demonstrated a combined occurrence of specific intestinal microbiome communities and disrupted TRP metabolism in various NDDs (9, 34). Therefore, we hypothesized that gut microbes might contribute to the abnormal TRP metabolism in NDDs.

Polyriboinosinic-polyribocytidylic acid (Poly I:C) is a class of double-stranded RNA synthetic analogs. Our research, along with previous studies, has demonstrated that prenatal Poly I:C exposure, mimicking infection in pregnant rodent females, can result in acute maternal immune activation and neurobehavioral changes in adolescent offspring (35–38). Thus, it provides a reliable MIA-induced NDDs model for investigating the potential pathological mechanism (39). While Poly I:C is a viral mimetic, the MIA it incites represents a systemic inflammatory event capable of disrupting multiple developmental pathways in offspring. Critically, prenatal Poly I:C exposure has been demonstrated to induce lasting alterations in the offspring’s gut microbiome composition, modeling dysbiotic states observed in neurodevelopmental disorders (40). Furthermore, it is a potent activator of the KYN pathway of TRP metabolism (41), a pivotal immunometabolic pathway implicated in neuroinflammation. However, the relationship between these two MIA-induced pathological events – gut microbiome dysbiosis and TRP metabolic disruption – remains poorly understood. Therefore, we hypothesize that prenatal viral-like MIA triggers parallel disruptions in the gut microbiome and TRP metabolism in offspring, and that these two pathological events are interrelated and collectively contribute to the observed neurodevelopmental abnormalities. To test this hypothesis, this study investigated the roles of TRP metabolism and gut microbiota in the progression of MIA-induced NDDs caused by prenatal Poly I:C exposure in female juvenile offspring, a rat model that previously showed deficits in both behaviors and the neuroimmune system (35, 36, 42, 43), considering that females should not be excluded from suffering from NDDs but are rarely included in animal studies.

## 2 Materials and methods

### 2.1 Animals and treatment

As shown in **Supplementary Figure S1A**, the prenatal Poly I:C exposure rat model was constructed following our previous reports (35, 36, 42, 43). In brief, sixteen specific pathogen-free (SPF) pregnant Sprague-Dawley rats were purchased from Sibeifu Biotechnology (Beijing, China) at gestation day (GD) 8. After one week of environmental adaptation, they were randomly assigned into two groups (n = 8) at GD15 (mid-late gestation). One group received an intraperitoneal (IP) injection of 5 mg/kg Poly I:C (Invivogen, Toulouse, France) dissolved in 0.2 mL of 1% phosphate buffered saline (PBS), while the second group received an equivalent volume of PBS. To reduce the abortion risk induced by invasive processes (such as peripheral blood collection), anal temperature and body weight changes at 0, 2, 4, 6, 8, 24, and 48h of post-injection were measured for Poly I:C-induced acute reaction monitoring (44, 45).

At PD 21, all offspring pups were weaned and housed in individually ventilated cages (IVCs) under environmentally controlled conditions (22 °C, light cycle from 07:00 to 19:00 and dark cycle from 19:00 to 07:00). Water and standard laboratory chow diet were freely available. To minimize litter effects, half of the female offspring from the same dam were randomly selected for this study (saline n = 16, Poly I:C n = 16), while all males and the rest of the female pups were transferred to a differently designed study (not published). Considering the social habits and cleanliness of the rat, each cage housed 5–6 animals from the same group. To minimize the effects of estrogen, all animals were housed in a single space. All experimental procedures in this study were approved by the Animal Ethics Committee, Fujian Maternity and Child Health Hospital, Fujian, China (2021KLRD638), and complied with the rules of Chinese Laboratory Animals – General Code of Animal Welfare (GB/T 42011-2022).

### 2.2 Behavioral tests

At PD 34-54, all animals underwent a series of behavioral tests, including the open field (OFT), three-chamber social test (TCST), elevated plus maze (EPM), and Morris water maze (MWM). Their behavioral performance was recorded and analyzed using the software Tracking Master v4.1.3 (Zhongshi Technology, Beijing, China). Before conducting the next behavioral test, all rats had at least two rest days. All tests were conducted between 8:30 and 17:00, alternated between groups.

#### 2.2.1 Open field test (PD 34-38)

The open field test was conducted to evaluate locomotor activity and anxiety-like behavior in a novel environment. Following established procedures (46), each rat was placed into the center of a black rectangular arena (100 cm × 100 cm × 40cm) for 8 minutes of free exploration. The average light intensity of the entire arena was 25 lux. The number of fecal pellets and the frequency of standing on their hind legs to explore were manually counted. The total distance moved, time spent in the arena or the center zone (50 cm × 50 cm square in the inner space), and the frequency of center zone entries were calculated by the software.

#### 2.2.2 Three-chamber social test (PD 40-44)

The three-chamber social test was conducted to assess social behavioral capabilities. The light intensity was set at 20–30 lux, and the experimental structure consisted of three chambers measuring 120cm × 40 cm × 40 cm. The test animals were placed in the central chamber with closed doors of the left and right partitions, allowing them to move freely for 10 minutes to acclimate to the environment. The test was conducted in two phases. Phase One: The stranger 1 (S1) rat of the same species and a similar age was randomly placed in a cage on one side, while the other cage remained empty. The doors were opened to allow the test rat to move freely for 10 minutes, and interaction behaviors with the S1 rat and the empty cage were recorded. Phase Two: The position of the S1 rat remained unchanged. The stranger 2 (S2) rat was placed into the previously empty cage, and the test rat was returned to the central chamber. During this process, visual contact between the test rat and the S2 rat was prevented. The doors were then opened to allow the test rat to move freely for 10 minutes, and interaction behaviors with both the S1 and S2 rats were recorded. The social index was calculated by T_S1_/(T_S1_ + T_empty_) or T_S2_/(T_S1_ + T_S2_).

#### 2.2.3 Elevated plus maze test (PD 47-48)

The elevated plus maze test was used to measure emotional behavior with a black plastic “cross” device elevated 60cm above the floor (47). The device consisted of 2 open arms (50 cm × 10 cm) and 2 closed arms (50 cm × 10 cm × 40 cm) with an open roof arranged around a central platform (10 cm × 10 cm). The light intensity was set at 100 lux in the open arms and central platform. Each rat was placed on the central platform facing an open arm for 5 minutes of free exploration. The frequency of open and closed arm entries (whole body and all four paws entries) and the duration spent in each arm were calculated. A rat was considered to be in the central platform zone if its head and front paws were on the central platform, with its body positioned in the closed arm.

#### 2.2.4 Morris water maze test (PD 50-54)

To evaluate spatial learning and memory, a Morris water maze experiment containing four consecutive days of fixed navigation trials and the subsequent probe trial on the fifth day was performed in a circular pool (150cm in diameter, 60cm in height) filled with water to 60% of its capacity. At the edge of the first quadrant of the pool, there was a black circular platform 12cm in diameter submerged in 1cm of water. During the first four consecutive days of fixed navigation trials, each rat was put into the pool from four different quadrants once per day. The swimming trajectories were recorded until the animal climbed onto the target platform within 60 seconds. Any rat that could not find or climb onto the platform within the allotted time was guided to the platform and allowed to stay there for 10–30 seconds before continuing with the next quadrant training. During the probe trial on the fifth day, the platform was removed, and each rat was put into the pool from the third quadrant, allowing them 60 seconds to search for the previously located platform. The latency time and the percentage of distance in the platform area during navigation and the time around the platform area during the subsequent probe trial were analyzed. To enhance the animals’ adaptability and the accuracy of the experiment, the pool was enclosed with a curtain, the light source was adjusted to avoid reflection in the water, and the water temperature was maintained at 24-26°C throughout the experiment.

### 2.3 Sample collection

Eight offspring from each group were randomly selected for both stool specimens and plasma collection (n = 8/group), and four and six whole brains from each group were randomly collected for immunohistochemical staining and expression analysis, respectively. To minimize the false positive (type I error) or effect size overestimation induced by litter effects, the animals selected for the same type of sample collection were from different dams (**Supplementary Figure S1B**). To avoid the potential effects of behavioral tests on the gut microbiota of the offspring, stool specimens were collected at PD 30, while both plasma and brain tissue were collected at PD 60 after euthanizing the rats with isoflurane anesthesia. For stool specimens, each rat was placed in a clean cage lined with a medical pad. Two fresh fecal pellets were collected into a cryopreservation tube by a sterile tweezer, with two tubes prepared for gut microbiota and non-targeted metabolomics analysis, respectively. For plasma collection, 5 mL of cardiac blood was centrifuged immediately at 3000 rpm at room temperature for evaluating peripheral TRP metabolism. Whole brains were dissected by surgical scissors. The brain tissues for immunohistochemical staining were immobilized in 4% paraformaldehyde and stored at room temperature. All other brain samples were immediately frozen in liquid nitrogen for 40min before being stored at -80 °C until use.

### 2.4 Immunohistochemical staining

Immunohistochemical staining targeting ionized calcium-binding adapter molecule 1 (Iba1) antibody was applied to evaluate the state of microglia in the PFC. Briefly, brain tissue containing the PFC was embedded in a wax block and sliced into 7-µm-thick sections using a rotary microtome (Leica, Wetzlar, Germany). After baking at 65°C for 30 minutes and dewaxing, the tissue sections were soaked in 100%, 95%, 80%, and 75% ethanol for 5 minutes each. The sections were then boiled for 15 minutes with citric acid for antigen retrieval and blocked with goat serum (ZLI-9056, ZSBG-BIO, Beijing, China) for 60 minutes. Following overnight incubation at 4°C with the Iba1 antibody (1:1000, #GTX100042, Genetex, Texas, USA), the slices were re-incubated by enzyme-labeled goat anti-rabbit IgG (1:500, ZENBIO, Sichuan, China) at 37°C for 20 minutes. Color development was performed with diaminobenzidine for 5 minutes, followed by re-staining with hematoxylin dye for 30 seconds. All images were captured with a microscope (Leica, Wetzlar, Germany) and analyzed by ImageJ (National Institutes of Health, Bethesda, USA). The size and density (cell numbers/mm^2^) of Iba1-positive cells were determined to evaluate the state of microglia.

### 2.5 Reverse transcription-qPCR

Reverse transcription quantitative polymerase chain reaction (RT-qPCR) was employed to measure the mRNA levels of microglia-activated markers in both the proinflammatory (M1) state (*Tnf-α, Cd86, Il-1β)* and anti-inflammatory (M2) state (*Cd206, Tgf-β2, Tgf-β3*; see **Supplementary Table S1** for details of Primer sequences) in the PFC. In brief, frozen brain tissue was cut into 500 µm coronal sections by a cryostat (Leica, Wetzlar, Germany) at -10.5 ± 1.5°C, and PFC tissue was collected following the rat brain atlas (48). The brain tissue was homogenized by a high-speed tissue grinder (Servicebio Technology, Wuhan, China) for total RNA extraction using a PureLinkTM RNA miniature kit (Invitrogen, Camarillo, USA). After being quantified and determining the quality with a NanoDrop^®^ 2000 (Thermo Fisher Scientific, Waltham, USA) and 1.5% agarose gel, respectively, 2 µg of RNA was reverse-transcribed into cDNA by a high-capacity cDNA reverse transcription kit (Thermo Fisher Scientific, Waltham, USA). RT-qPCR was run on the Quant Studio™ RT-qPCR system (Thermo Fisher Scientific, Waltham, USA) under the following conditions: 95°C for 10 minutes, followed by 40 cycles at 95°C for 15 seconds and 60°C for 1 minute. All samples were measured in duplicate using the SYBR™ Green PCR master mix (Thermo Fisher Scientific, Waltham, USA). The primers used are listed in **Supplementary Table S1**. The relative mRNA expression of each target gene was normalized to both β-actin and Gapdh. The normalized values were calculated using the 2-ΔΔCT method.

### 2.6 Western blot

Western blot was applied to analyze the key proteins or enzymes of the TRP-KYN pathway in brain regions, including Lat1 (transporter of both TRP and KYN across the BBB), Ido1 (a key enzyme directing TRP toward the KYN pathway), KatII (a key enzyme of KYN toward KYNA pathway) and Kmo (a key enzyme of KYN toward QA pathway). In brief, the PFC tissue was lysed in RIPA lysate (EpiZyme, Shanghai, China) containing 1% Protease inhibitors (EpiZyme, Shanghai, China) for 30 minutes. The protein concentration was determined using a bicinchoninic acid (BCA) protein quantitative kit (EpiZyme, Shanghai, China). The protein was then separated by SDS-PAGE (Biosharp, Anhui, China) and transferred to a nitrocellulose (NC) membrane (Merck Millipore, Massachusetts, USA). The membranes were incubated overnight at 4°C with the following primary antibodies: anti-Lat1 (1:1000, Abconal, Shanghai, China), anti-Ido1 (1:500, Abconal, Shanghai, China), anti-Kmo (1:1000, Proteintech Group, Wuhan, China), anti-KatII (1:2000, Abconal, Shanghai, China). The next day, the membrane was incubated with secondary antibodies (1:3000, Cell Signaling Technology, Massachusetts, USA). After washing and adding the exposure solution, the membranes were photographed and analyzed using ImageJ (National Institutes of Health, Bethesda, USA).

### 2.7 Enzyme-linked immunosorbent assay

Enzyme-linked immunosorbent assay (ELISA) was employed to quantify the plasma concentrations of tryptophan (TRP, Cloud-Clone, Houston, USA), kynurenine (KYN, Enzyme-Linked Biotechnology, Shanghai, China), and serotonin (5-HT, ZCIBIO, Shanghai, China) to assess the direction of the TRP metabolic pathway in the peripheral circulation.

To avoid potential interference from hemolysis, two samples were excluded owing to severe hemolysis (one from the Poly I:C group and one from the saline group; see **Supplementary Figure S1B**). Each sample was tested in duplicate. The optical density (OD) of each well was measured using a microplate reader (Tecan, Männedorf, Switzerland) at a wavelength of 450 nm within 15 minutes of the experiment. The concentrations were determined based on the standard concentration gradient curve.

For the TRP assay, an equal amount of detection solution A was added immediately to the well filled with 50 μL of the sample or diluted standard solutions of different concentrations. After incubating at 37°C for 1 hour and washing three times, 100 μL of test solution B was added to each well and incubated for 30 minutes. The wells were then washed five times and incubated with 90 μL substrate solution for 20 minutes in a dark room at 37°C, before adding 50 μL of termination solution. For the KYN assay, a 50 μL sample or diluted standard solution of different concentrations was added to the wells, followed by 100 μL of horseradish peroxidase (HRP). After incubating at 37°C for 1 hour, wells were washed five times, and 50 μL each of substrate A and substrate B was added. The wells were incubated for 15 minutes in a dark room at 37°C before adding 50 μL of termination solution. The procedure for the 5-HT assay was similar to that for KYN. Ratios of KYN/TRP and 5-HT/TRP were used to evaluate the conversion of TRP to KYN and the serotonin pathway, respectively (49).

### 2.8 Liquid chromatography-mass spectrometry/mass spectrometry

Non-targeted metabolomics was analyzed using liquid chromatography-mass spectrometry/mass spectrometry (LC-MS/MS). Briefly, metabolite extraction was conducted by adding 50 ± 5 mg of fecal sample to 400 µL of extraction solution (methanol:water = 4:1, v/v) containing 0.02 mg/mL of internal standard (L-2-chlorophenylalanine). This was followed by freezing, grinding, ultrasonication, and centrifugation. The supernatant was then analyzed using a UHPLC-Q Exactive HF-X system (ThermoFisher Scientific Inc., USA). Detailed analysis conditions and parameters were set according to the reference (50). To monitor analysis stability, a pooled quality control (QC) sample, prepared by mixing equal volumes of all samples, was injected every 10 samples. The chromatographic separation was then carried out under the following conditions: A volume of 3 μL of the extracted sample was injected and separated on a HSS T3 column (100mm × 2.1mm i.d., 1.8 μm). The mobile phases consisted of (A) water:acetonitrile = 95:5 (v/v) with 0.1% formic acid and (B) acetonitrile:isopropanol:water = 47.5:47.5:5 (v/v/v) with 0.1% formic acid. The separation was achieved using a gradient elution at a constant flow rate of 0.40 mL/min. The sample mass spectrometry signal acquisition was conducted in positive and negative ion scanning mode, with a mass scanning range of 70–1050 m/z. The sheath gas flow rate was 50psi, the auxiliary gas flow rate was 13psi, the auxiliary gas heating temperature was 425°C, the positive mode ion spray voltage was set at 3500V, the negative mode ion spray voltage was set at -3500V, the ion transfer tube temperature was 325°C, and the normalized collision energy was 20, 40, 60V in a cyclic collision energy mode. The resolution of the first mass spectrometry was 60,000, and the resolution of the second mass spectrometry was 7,500. Data were collected in DDA mode. Raw data pre-processing was performed using Progenesis QI software (Waters Corporation, Milford, USA). After removing the internal standard peak and known false positive peaks (noise, column bleed, and derivatized reagent), the filtered data were identified by matching against the HMDB (http://www.hmdb.ca/), MetLin (https://metlin.scripps.edu/), and the Majorbio database. The relative standard deviation (RSD) of the total ion current (TIC) peak area across QC samples was used as the primary indicator of mass spectrometry system stability. Analytical runs were considered stable and the data reliable if more than 80% of the QC samples exhibited an RSD below 30% for the TIC in both positive and negative ionization modes.

### 2.9 16S small subunit ribosomal RNA sequencing

Gut microbial composition and diversity were analyzed using 16 small subunit ribosomal RNA (16S rRNA) sequencing. Briefly, total microbial genomic DNA was extracted from a 100 ± 5mg stool specimen using a Mag-Bind Stool DNA Kit (Omega Bio-Tek, Georgia, USA) following the manufacturer’s protocol. The quality and concentration of the DNA were measured using 1.0% agarose gel electrophoresis and a NanoDrop^®^ ND-2000 spectrophotometer (Thermo Fisher Scientific Inc., USA). The qualified DNA was then analyzed in triplicate by amplifying the hypervariable region V3-V4 of the bacterial 16S rRNA gene using primers (5’-ACTCCTACGGGAGGCAGCAG-3’ and 5’-GGACTACHVGGGTWTCTAAT-3’) (51) on an ABI GeneAmp^®^ 9700 PCR thermocycler (Applied Biosystems, Carlsbad, USA). The purified PCR products were sequenced using paired-end sequencing on an Illumina PE300/PE250 platform (Illumina, San Diego, USA) according to standard protocols by Majorbio Bio-Pharm Technology Co. Ltd. (Shanghai, China).

### 2.10 Data analysis

#### 2.10.1 Bioinformatic analysis of multi-omics datasets

The clean data matrix obtained from both the metabolomics study and gut microbiome analysis was uploaded to the Majorbio Cloud platform (https://cloud.majorbio.com/page/project/overview.html) for further bioinformatic analysis. For non-targeted metabolites, before the subsequent analysis using the R package “ropls” (Version 1.6.2), metabolic features below the lower limit of quantification or those with minimal values were normalized to the total sum. Variables in QC samples with a RSD > 30% were excluded and log10 transformed. Principal coordinate analysis (PCoA) was utilized to investigate the similarity in sample community composition. Orthogonal partial least squares discriminant analysis (OPLS-DA) was applied to construct the regression model between metabolite expression and sample treatment characteristics. An intercept value less than 0.05 or an upward trend of the regression line indicates that the model is robust and reliable without overfitting during model stability evaluation. Differential metabolites between groups were analyzed and displayed as volcano maps. The biological function of differential metabolites was analyzed using Python for Kyoto Encyclopedia of Genes and Genomes (KEGG) pathway enrichment analysis.

For the 16S rRNA sequencing, raw FASTQ files were de-multiplexed using an in-house Perl script, quality-filtered with Fastp version 0.19.6 (52), and merged using FLASH version 1.2.11 (53). The optimized sequences were processed using the UPARSE algorithm with a 97% sequence similarity cutoff to cluster taxonomic units (54). Taxonomic classification from domain to species level was performed using RDP Classifier version 2.13 against the 16S rRNA gene database (e.g., SILVA v138) with a confidence threshold of 0.7 (55). Alpha diversity indices, including the Chao, Ace, Shannon, and Simpson indices, were calculated using Mothur v1.30.2 (56). Rarefaction curves based on the Sobs and Shannon indices were applied to assess sequencing depth. The similarity among the microbial communities in different samples was determined by PCoA based on Bray-Curtis dissimilarity using the Vegan v2.4.3 package. The Wilcoxon rank-sum test was used to analyze the beta diversity between the two different groups. According to the reference (57), the original gut microbiota health index (GMHI), was derived from shotgun metagenomic data. In the present study, we adapted this health index by calculating it based on the relative abundance of the bacterial genera that were used in the original GMHI formulation. A Wilcoxon rank-sum test was then used to determine whether prenatal Poly I:C treatment induced intestinal dysbiosis based on GMHI. The top 15 significant genera were determined by a Wilcoxon rank-sum test. The dominant bacteria at the genus level in each group were identified using linear discriminant analysis (LDA) effect size (LEfSe) (http://huttenhower.sph.harvard.edu/LEfSe) with an LDA score > 2.5 and *p <*0.05 *(* 58). The correlation analysis between the dominant differential genus and gut metabolites or behavioral indicators was performed using Python (version 1.0.0) and the results are visualized as a clustering heat map.

#### 2.10.2 Statistical analysis of data for all other experiments

Data were analyzed and visualized using GraphPad Prism 8.0.2 (GraphPad Software Inc., California, USA). Outliers were identified and removed using Boxplot on SPSS 26.0 (IBM, New York, USA). Acute reaction evaluations (anal temperature and weight) and fixed navigation trials from the Morris water maze test were analyzed using a two-way repeated ANOVA (Treatment × Time as repeated measures). Paired t-test (for normally distributed data) or Wilcoxon signed-rank test (for non-normally distributed data) were used to compare changes at adjacent time points within groups. For all other experimental data, group differences, and correlations were analyzed using the Student’s t-test and Pearson’s test, respectively, for normally distributed data; otherwise, the nonparametric Mann-Whitney U test and Spearman test were applied. *P <*0.05 was considered statistically significant. The results were presented as mean ± standard error of the mean (SEM). Before performing statistical analysis, data from the same dam during the behavior tests were averaged, and one rat in the Saline group was excluded due to accidental death during the experimental period.

## 3 Result

### 3.1 Maternal acute reactions and offspring behavior changes induced by Poly I:C administration

#### 3.1.1 Maternal acute reactions

**Figure 1:**
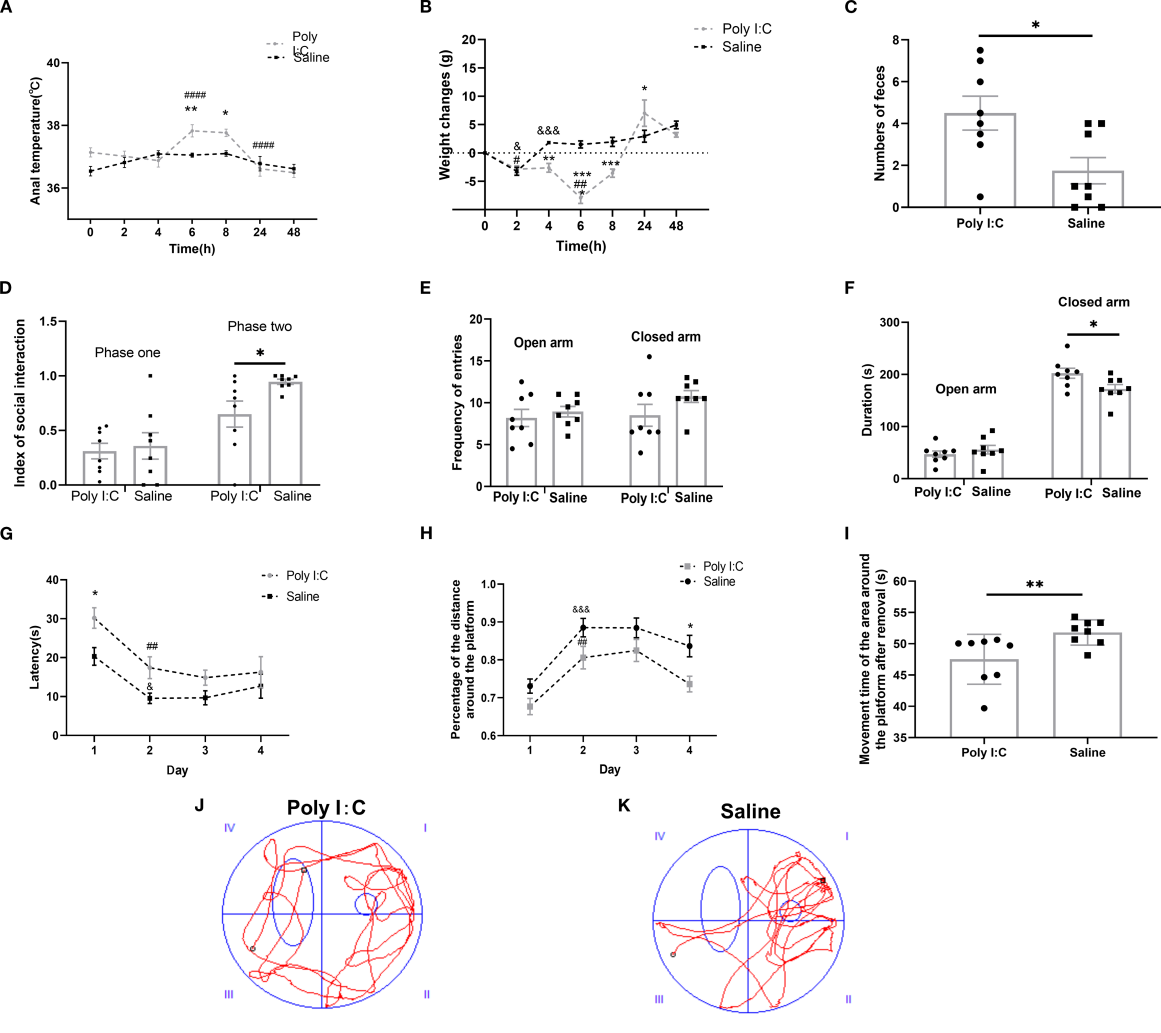
Maternal Poly I:C exposure led to an acute pathogenic response in dams and behavioral abnormalities in juvenile female offspring. **(A)** Anal temperature; **(B)** Body weight changes of dams after injection; **(C)** Numbers of fecal pellets during the open field test; **(D)** Index of social interaction; **(E)** Frequency of arms entries in the elevated plus maze test; **(F)** Duration of arms; **(G-K)** Morris water maze test: **(G)** Latency (s) during Fixed navigation phase; **(H)** Percentage of distance in the target quadrant; **(I)** Time spent in the target quadrant (s) during the probe trial phase; Swimming trajectory of rats from the **(J)** Poly I:C and **(K)** Saline groups; * *p <*0.05, ** *p <*0.01, *** *p <*0.001, **** *p <*0.0001, Poly I:C *vs*. Saline; # *p <*0.05, ## *p <*0.01, #### *p <*0.0001, comparison of adjacent time points within the Poly I:C group; & *p <*0.05, &&& *p <*0.001, comparison of adjacent time point within Saline group. Data are presented as mean ± SEM. Sample size: n = 8.

There were significant main effects of both “Time” (F_6,98_ = 8.933, *p <*0.0001) and “Treatment” (F_1,98_ = 8.201, *p* =0.0051), and an interaction effect between these two factors (F_6,98_ = 3.472, *p* =0.0037) on the anal temperature. At both 6th and 8th hour, Poly I:C rats exhibited a significantly higher anal temperature compared with controls (*p <*0.01 and < 0.05, respectively). Meanwhile, the anal temperature of Poly I:C rats showed a significantly higher at the 6^th^ hour compared with the 4^th^ hour, but a significantly lower at the 24^th^ hour compared with the 8^th^ hour (both *p <*0.0001). No significant difference in anal temperature was observed in Saline rats throughout the monitoring(all *p* > 0.05) (**Figure 1A**).

Similarly, for the body weight changes, two-way ANOVA showed that there were significant main effects of both “Time” (F_6,98_ = 25.69, *p <*0.0001) and “Treatment” (F_1,98_ = 24.96, *p <*0.0001), and there was a significant interaction effect between these two factors (F_6,98_ = 12.44, *p <*0.0001). Compared with the former monitoring point, the rats with saline treatment had a significant weight loss at the 2^nd^ hour (*p <*0.05), but a significant body weight gain at the 4^th^ hour (*p <*0.001) and no change in the subsequent monitoring (all *p* > 0.05). For the rats with Poly I:C treatment, significant body weight loss was observed at both the 2^nd^ (*p <*0.05) and 6^th^ hour (*p <*0.01). Therefore, compared with Saline, there was a significant difference in the value of body weight changes between the rats of Poly I:C and Saline at the monitoring points of 4^th^ hour, 6^th^ hour, 8^th^ hour, and 24^th^ hour, respectively (*p <*0.01, *p <*0.0001, *p <*0.001 and *p <*0.05, respectively; **Figure 1B**). These results provided evidence that IP injection of 5 mg/kg Poly I:C at GD15 effectively induced acute pathogenic response in pregnant rats.

#### 3.1.2 Behavioral changes in offspring

In the open field test, there were no significant differences between Poly I:C rats and controls in rearing frequency; total distance moved in the arena or central zone; accumulated time spent in the arena or central zone; or frequency of center zone entries (all *p >* 0.05, **Supplementary Table S2**). However, offspring rats with prenatal Poly I:C exposure showed a significant increase in fecal pellet numbers compared to controls (*U_8,8_* = 12, *p* =0.035; **Figure 1C**). These data indicate elevated anxiety-like behavior in Poly I:C rats. The increased number of fecal pellets expelled may be indicative of elevated anxiety-like behavior, although a contribution from altered gastrointestinal function due to the Poly I:C-induced dysbiosis cannot be ruled out.

In the three-chamber social test, there was no significant difference in the social index between the two groups during phase one (0.31 ± 0.07 *vs*. 0.36 ± 0.12, *p* > 0.05; **Figure 1D**). In contrast, during phase two, Poly I:C rats exhibited a significantly lower social index than that of Control (0.74 ± 0.09 *vs*. 0.95 ± 0.02, *p <*0.05; **Figure 1D**). These data indicated a deficiency in social ability in Poly I:C rats.

In the elevated plus maze test, while there were no significant differences between Poly I:C rats and controls in frequency of both the open and closed arms entries, or duration staying in open arms (all *p >* 0.05; **Figures 1E, F**), there was a significant increase of closed arm duration in the rats of Poly I:C (t = 2.365, df = 14, *p* =0.033, **Figure 1F**). These data indicate elevated anxiety-like behavior in female juvenile offspring rats.

During the four-day fixed navigation trials of the Morris water maze test, there were significant main effects of both “Day” (F_3,55_ = 10.840, *p <*0.0001) and “Treatment” (F_1,55_ = 13.050, *p* =0.0007), with no significant interactions (F_3,55_ = 0.572, *p* =0.636) between the two factors for the “latency” parameter. Rats with prenatal Poly I:C exposure exhibited significantly longer latency on day 1 (*p <*0.05; **Figure 1G**). Compared with Day 1, significantly shorter latency was observed on Day 2 in both Poly I:C rats (*p* =0.010), and Saline rats (*p* =0.022). There were significant main effects of both “Day” (F_3,56_ = 15.32, *p <*0.0001) and “Treatment” (F_1,56_ = 17.09, *p <*0.001), with no significant interactions (F_3,56_ = 0.35, *p* ≥ 0.05) between the two factors for “Percentage of the distance around the platform”. The percentage of the distance around the platform for rats in both groups on the second day was significantly higher than those on the first day (Poly I:C group: *p <*0.01; Saline group: *p <*0.001). However, the percentage of distance spent in the platform zone for rats in the Poly I:C group on the fourth day was significantly lower than the control group (*p <*0.05; **Figure 1H**).

During the probe trial, Poly I:C rats exhibited aimless swimming trajectories (**Figure 1J**), while control rats concentrated their swimming around the platform area (**Figure 1K**). Compared with Control, the time spent in the target quadrant was significantly less in the Poly I:C group (*U_8,8_* =67, *p* =0.007; **Figure 1I**). These results suggested that maternal Poly I:C exposure caused deficits in spatial learning and memory in female juvenile offspring.

### 3.2 Alterations in microglial immunoreactivity and the TRP-KYN pathway suggests a potential shift towards the QA direction in the PFC of juvenile female offspring

Multiple lines of evidence support the association between neurobehaviors and executive functions mediated by the PFC (59–61). Within the PFC, microglia are active players in complex neurodevelopmental programs. They interact with neurons, provide trophic support, and respond to cytokine and metabolic signals from the local neural environment (62). To elucidate the pathological mechanisms underlying the learning, memory deficits, and anxiety induced by maternal Poly I:C exposure, this study analyzed molecular changes in microglia within the PFC.

**Figure 2:**
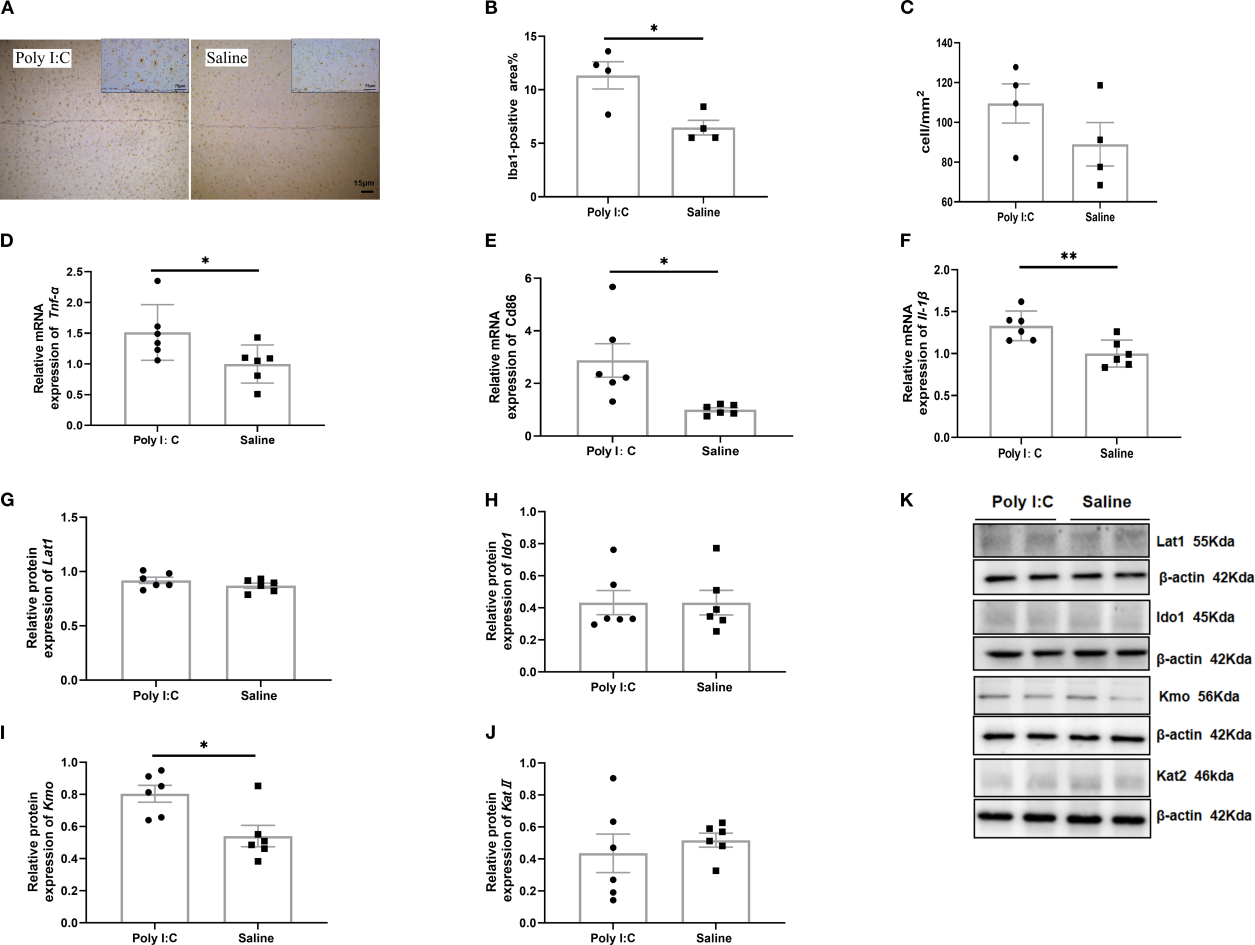
Microglia immunohistochemical staining, gene expression of activated markers, and protein expression of the TRP-KYN pathway in the prefrontal cortex of rats from the Poly I:C and Saline groups. **(A)** Staining image (20x); **(B)** Positive area%; **(C)** Number of Iba1-positive cells per mm² (n = 4/group). Relative mRNA level of **(D)** *Tnf-α*, **(E)** *CD86*, **(F)** *Il-1β* (n = 6/group). Protein levels of **(G)** Lat1, **(H)** Ido1, **(I)** Kmo, **(J)** KatII in the TRP-KYN pathway. **(K)** Example images of Western blot. The original Western blot images are shown in **Supplementary Figure S3**. Data are expressed as mean ± SEM; * *p <*0.05, ** *p <*0.01.

Microglial immunoreactivity analysis showed that the size of Iba1-positive cells in rats from the Poly I:C group was significantly larger than that in the control group (t = 3.364, df = 6, *p* =0.015; **Figures 2A, B**), but there was no significant difference in the density of these cells (t = 1.394, df = 6, *p* =0.213; **Figures 2A, C**). Meanwhile, prenatal Poly I:C exposure upregulated mRNA expression of M1-related microglial markers, including *Tnf-α* (t = 2.297, df = 10, *p* =0.044; **Figure 2D**), *Cd86* (t = 2.909, df = 10, *p* =0.016; **Figure 2E**), and *Il-1β* (t = 3.389, df = 10, *p* =0.007; **Figure 2F**), but did not affect the expressions of M2-related markers, *Tgf-β2, Tgf-β3* and *Cd206* (**Supplementary Table S3**). These findings suggested that maternal Poly I:C stimulation led to microglia activation toward a pro-inflammatory state in the PFC of juvenile offspring rats.

In addition, Western blot analysis of key proteins and enzymes associated with TRP-KYN pathway revealed that, while no significant differences were observed in the relative expression levels of Lat1, Ido1, or KatII in the PFC between Poly I:C rats and saline-treated controls (all *p* > 0.05; **Figures 2G, H, J, K**), a significant increase in Kmo protein levels was detected in the Poly I:C group (t = 3.098, df = 10, *p* =0.011; **Figures 2I, K**). Considering that Kmo is predominantly expressed in microglia (63), these findings suggested that the TRP-KYN metabolic pathway may shift toward the neurotoxic QA direction in the PFC of juvenile female offspring exposed to Poly I:C.

### 3.3 Promoted peripheral TRP metabolism activation through the KYN pathway in juvenile female offspring

**Figure 3:**
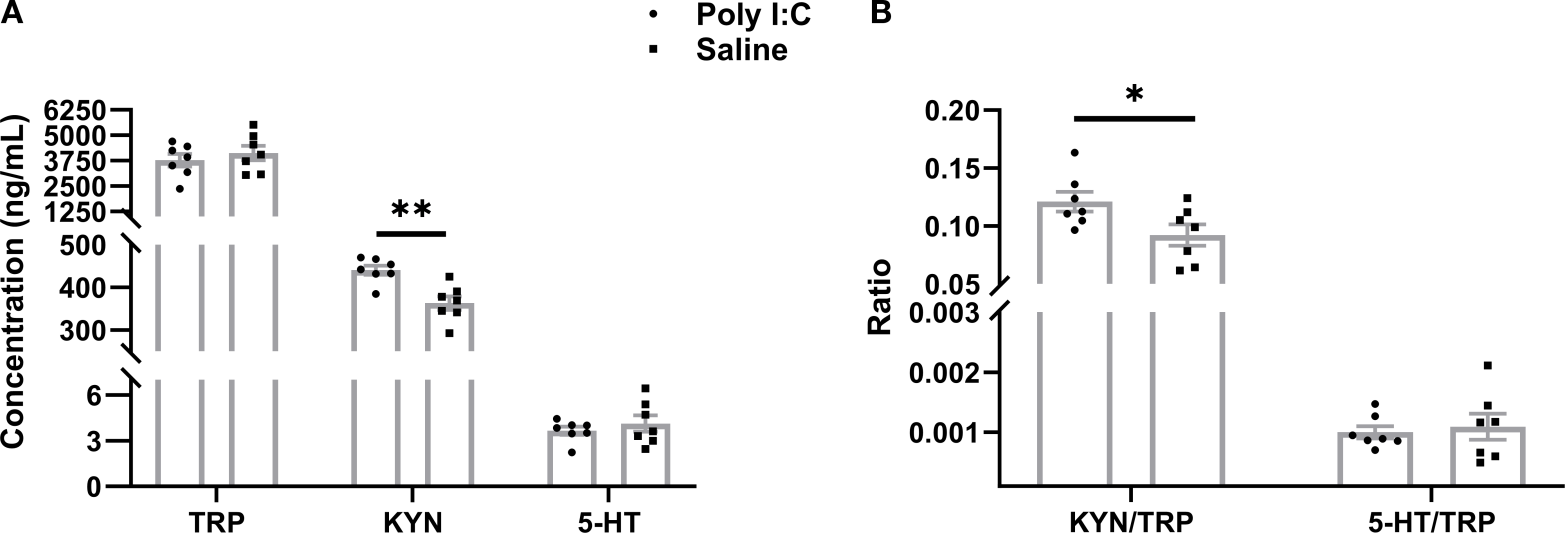
ELISA analysis of peripheral metabolites in TRP metabolism. **(A)** Concentration of TRP, KYN, and 5-HT (ng/mL), **(B)** Ratio of KYN/TRP and 5-HT/TRP. Data are expressed as mean ± SEM, n = 8/group (one sample from each group was excluded due to severe hemolysis); * *p <*0.05, ** *p <*0.01.

In plasma, there were no significant differences in the average levels of TRP (t = 0.781, df = 12, *p* =0.450) or 5-HT (t = 0.797, df = 12, *p* =0.450) between the Poly I:C and Saline groups. However, Poly I:C rats showed a significant increase in KYN concentration compared with controls (t = 4.012, df = 12, *p* =0.002; **Figure 3A**). Meanwhile, while the 5-HT/TRP ratio did not differ significantly between the two groups (t = 0.386, df = 12, *p* =0.706), the KYN/TRP ratio was significantly higher in rats with prenatal Poly I:C treatment (t = 2.312, df = 12, *p* =0.039) (**Figure 3B**). These results suggest that prenatal Poly I:C intervention promotes peripheral TRP metabolism through the KYN pathway in juvenile female offspring.

### 3.4 Intestinal TRP metabolism dysregulation associated with gut microbiota dysbiosis

#### 3.4.1 Intestinal TRP metabolism dysregulation in juvenile female offspring

**Figure 4:**
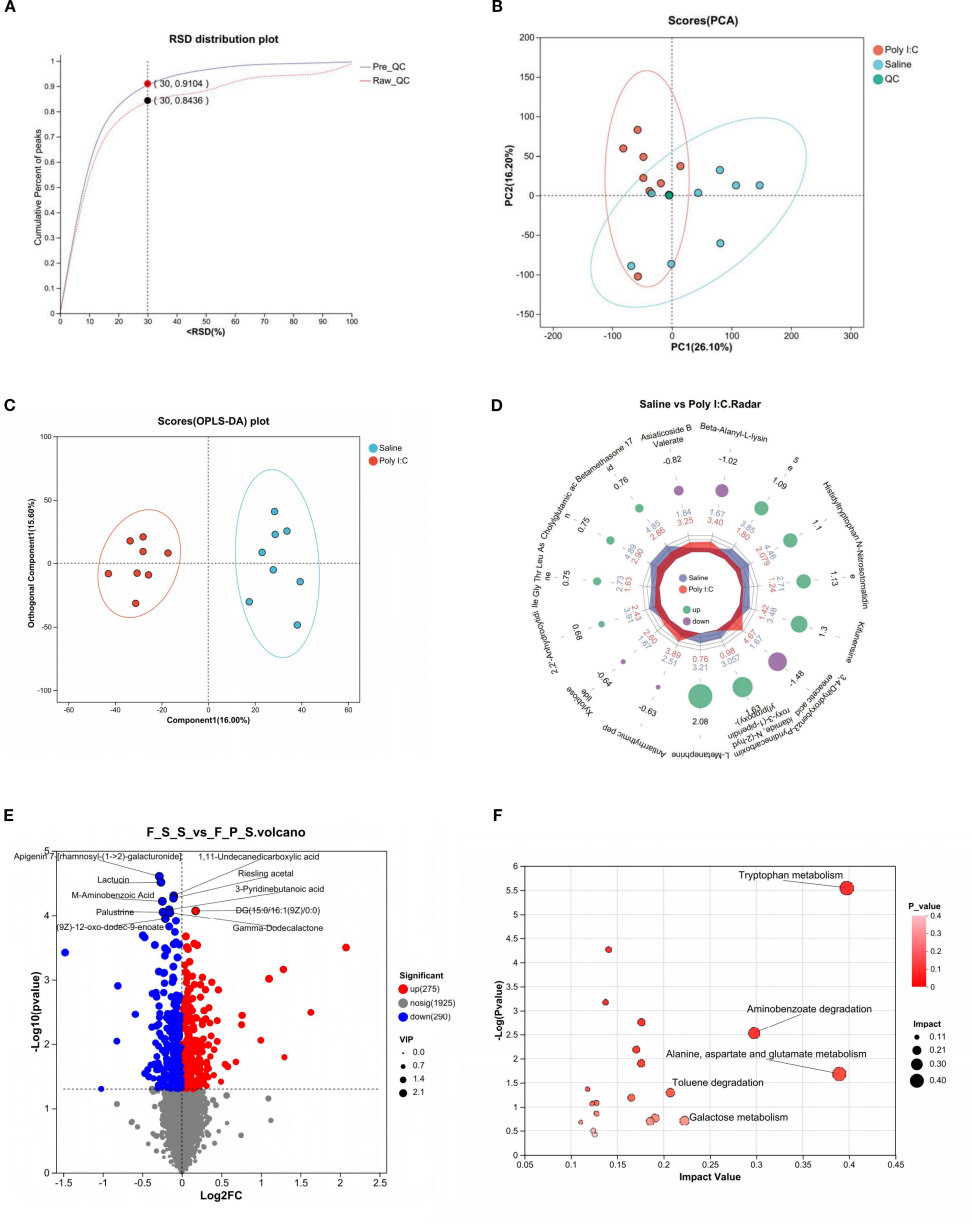
Intestinal metabolite analysis of female juvenile offspring rats with prenatal Poly I:C exposure. **(A)** RSD distribution plot; **(B)** PCA analysis; **(C)** OPLS-DA analysis; **(D)** Radar chart; **(E)** Volcano map of differential metabolites; “Up” indicates metabolites that are increased in the Control group relative to the Poly I:C group, while “Down” indicates metabolites increased in the Poly I:C group relative to the Control. **(F)** Topology analysis of bubble plot based on KEGG; n = 8/group; Data are expressed as mean ± SEM; * *p <*0.05, ** *p <*0.01, *** *p <*0.001.

It indicates that the analytical system exhibited excellent stability (**Figure 4A**). In the PCA, the QC samples were highly clustered near the center, reflecting a high degree of clustering among the samples and confirming the stability of the data (**Figure 4B**). The OPLS-DA model revealed distinct clustering of the two groups (**Figure 4C**). Furthermore, the major discriminating metabolites responsible for this separation were visualized using a radar chart (**Figure 4D**). Differential analysis showed a total of 290 metabolites were significantly increased, while 275 were significantly decreased (**Figure 4E**). KEGG pathway enrichment analysis of the differential metabolites revealed that “Tryptophan metabolism” was one of the most significantly enriched pathways (**Figure 4F**). The main metabolites involved in TRP metabolism are presented in **Table 1**. A total of 7 metabolites were identified through the TRP metabolic pathway. Among them, compared with the control group, the abundance of L-Tryptophan (L-TRP), Indole-3-Acetamide (IAM; a downstream metabolite of indole), N-formylanthranilic acid (NFAA, downstream metabolites of KYN), and 0N’-Formylkynurenine (NFK, downstream metabolites of KYN) were significantly increased in the Poly I:C group (all *p <*0.05). Conversely, the levels of Tryptophol (TOL, the metabolite of indole), N-acetyl-N-formyl-5-methoxykynuramine (AFMK, the metabolite of 5-HT), and QA (a metabolites of KYN) were significantly decreased (TOL: *p <*0.01; AFMK: *p <*0.05; QA: *p <*0.05). These results suggested that prenatal Poly I:C intervention induced intestinal TRP metabolism dysregulation in juvenile female offspring.

**Table 1:** Differentially abundant intestinal tryptophan metabolites in the Poly I:C group compared to the Saline group.

Metabolite	FC	m/z	FDR	Mode	Regulate	*p*_value
L-Tryptophan	0.96	205.10	0.15	pos	↑	0.014
Tryptophol	1.27	203.12	0.14	pos	↓	0.005
Indole-3-Acetamide	0.84	175.09	0.20	pos	↑	0.041
AFMK	1.15	265.12	0.20	pos	↓	0.041
Quinolinic Acid	1.13	168.03	0.17	pos	↓	0.024
N-formylanthranilic acid	0.97	164.03	0.13	neg	↑	0.024
N’-Formylkynurenine	0.95	271.05	0.13	neg	↑	0.024

The arrow “↑” indicates a significant increase in the Poly I:C group compared to the Saline group; “↓” indicates a significant decrease. FC, fold change; m/z, mass-to-charge ratio; FDR, false discovery rate.

#### 3.4.2 Intestinal dysbacteriosis in female offspring, with a focus on species associated with TRP metabolism

In 16S rRNA sequencing, a total of 983,665 optimized sequences with a length of 415,338,670 bp were obtained. The average optimized sequence in each sample was 61,479 ± 1635, and the average length per optimized sequence was 422.50 ± 0.93 bp. After clustering using UPARSE 11 at a 97% sequence similarity level, a total of 5222 taxonomic clusters were obtained from all optimized sequences (**Supplementary Table S4**). Acknowledging the development of more precise methods, we have also processed the raw sequences using the DADA2 plugin within QIIME2 to resolve Amplicon Sequence Variants (ASVs). This ASV table, provided in **Supplementary File 2**, offers a higher-resolution view of the microbial communities and is presented to support future in-depth and comparative analyses by the research community.

**Figure 5:**
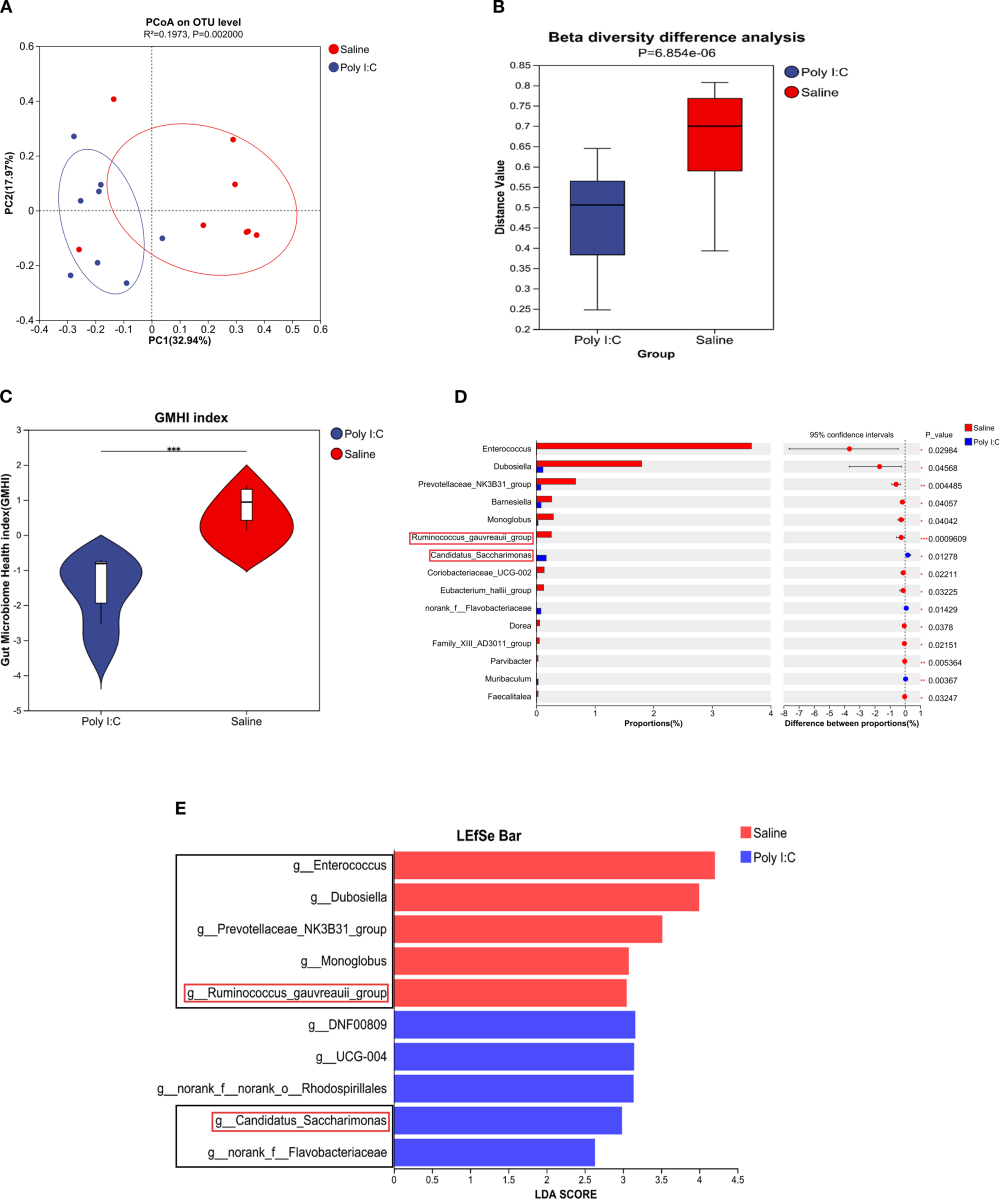
Profile of gut microbial community in female juvenile offspring rats with prenatal Poly I:C treatment. **(A)** PCoA analysis; **(B)** β diversity difference analysis; **(C)** GMHI index, **(D)** Differential abundance of microbial genera, and **(E)** LEfSe analysis. Abbreviations: GMHI, gut microbiota health index; PCoA, principal coordinate analysis; LEfSe, linear discriminant analysis (LDA) effect size. n = 8/group; Data are expressed as mean ± SEM; *** *p <*0.001; The black box highlights the main content.

Although there were no significant differences in alpha diversity indices (including Ace, Chao, Shannon, and Simpson) between the Poly I:C and Saline rat groups, rarefaction curves based on the Sobs index and Shannon indices gradually plateaued (**Supplementary Figure S2**), indicating sufficient sequencing depth. PCoA analysis on Bray-Curtis dissimilarity revealed a significant difference in gut microbial community distribution between Poly I:C-treated rats and control (R^2^ = 0.197, *p <*0.01; **Figure 5A**). The Wilcoxon rank-sum test further confirmed a significant difference in β diversity between the Poly I:C and Saline groups (*p <*0.0001; **Figure 5B**). To ensure that this difference was not driven by potential confounders, we addressed factors including cage and dam effects both experimentally and statistically. Specifically, animals were randomly caged, and statistical models for beta-diversity analysis included “cage” as a random effect to adjust for the non-independence of samples from the same cage. Additionally, the GMHI index was significantly lower in the Poly I:C rats than that of the control (*p <*0.001; **Figure 5C**), suggesting that prenatal Poly I:C treatment induced intestinal dysbacteriosis in juvenile female offspring. As shown in **Figures 5D, E**, the dominant differential genera identified in the control group were *Enterococcus*, *Dubosiella*, *Prevotellaceae*_NK3B31_group, *Monoglobus*, and *Ruminococcus gauvreauii*_group, while Candidatus *Saccharimonas* and norank_f_Flavobacteriaceae were dominant in the Poly I:C group. These bacteria may play a key role in the onset and progression of the disease.

#### 3.4.3 Correlation analysis

To further evaluate the association between dominant differential genera and TRP metabolites or abnormal behaviors induced by prenatal Poly I:C exposure, a correlation analysis was performed.

**Figure 6:**
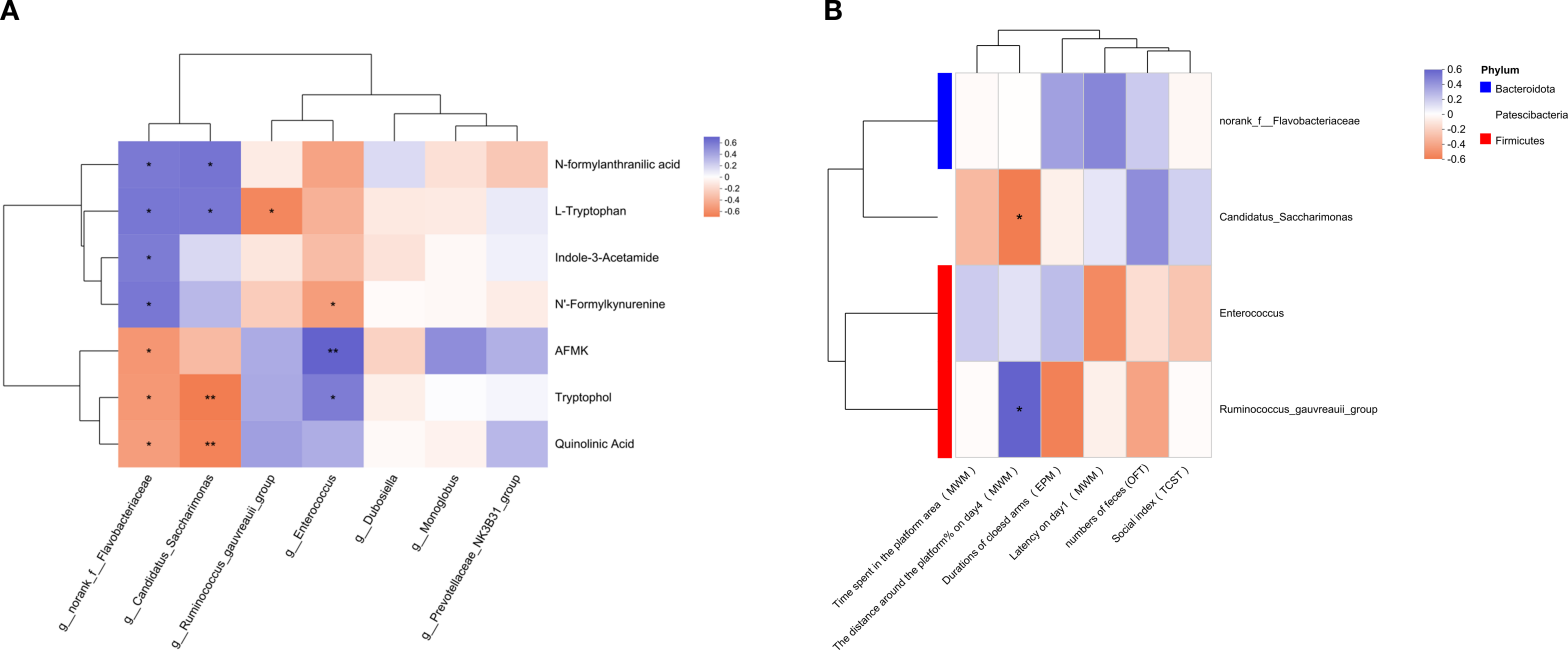
Relationship of significant changes of parameters among different experiments. Correlation analysis in **(A)** gut metabolites linking to TRP metabolism with intestinal microbiota of genus level, **(B)** intestinal microbiota of genus level with behavioral changes. The correlation R values were presented in different colors, with purple representing a positive correlation and orange denoting a negative correlation. * *p <*0.05, ** *p <*0.01, *** *p <*0.001. The red box identifies the main content.

As shown in **Figure 6A**, four dominant differential genera were significantly associated with TRP pathway metabolites, including *Enterococcus*, *Ruminococcus gauvreauii*_group, Candidatus *Saccharimonas*, and norank_f_Flavobacteriaceae. Among these, norank_f_Flavobacteriaceae exhibited significant correlations with all differential TRP metabolites (all *p <*0.05). Candidatus *Saccharimonas* was positively correlated with the abundance of L-TRP and NFAA (both *p <*0.05) and negatively correlated with QA and TOL (both *p <*0.01). The *Ruminococcus gauvreauii*_group was negatively correlated with L-TRP (*p <*0.05). *Enterococcus* was negatively correlated with NFK (*p <*0.05) but positively correlated with AFMK and TOL (AFMK: *p <*0.01; TOL: *p <*0.05).

As shown in **Figure 6B**, the number of feces from the OFT, the social index from the TCST, the duration of the closed arm in the EPM, the latency in the MWM (training period) or the time spent in the platform area (test period) showed no significant correlation with all the dominant differential genera (all *p* > 0.05). However, the percentage of distance in the target quadrant on the fourth day of the MWM in the training period exhibited a significant negative correlation with Candidatus *Saccharimonas* (*p <*0.05), while a significant positive correlation with the *Ruminococcus gauvreauii*_group (*p <*0.05).

## 4 Discussion

In the present study, we investigated the effects of TRP metabolism and gut microbiota on the neuropathological and behavioral changes in an MIA-induced NDDs model constructed by prenatal Poly I:C exposure. In addition to the acute pathogenic response in dams and the elevated anxiety-like behaviors and social deficiency in offspring, which are consistent with our previous reports (36), a Morris water maze test showed deficits in spatial learning and memory in the juvenile female rats with prenatal Poly I:C exposure (**Figure 1**). The microglial immunoreactivity analysis in the PFC region revealed a significant increase in the Iba1-positive area (**Figure 2B**). Parallel to these changes, the upregulation of Kmo (predominantly expressed in microglia) (**Figure 2K**) suggests long-term elevated neuroinflammation induced by prenatal Poly I:C exposure, potentially linked to a shift in the TRP-KYN pathway toward the QA direction. Importantly, maternal Poly I:C exposure promoted peripheral circulating TRP-KYN metabolism, indicated by significant increases in both KYN concentration and the KYN/TRP ratio in the plasma (**Figure 3**). Fecal multi-omics analysis showed that TRP digestion and catabolism in the intestine were disturbed in Poly I:C rats (**Figure 4F**). Furthermore, there was a significant decrease in gut bacteria diversity and changes in dominant bacterial species associated with TRP metabolism in Poly I:C rats (**Figure 5**). Notably, correlations were observed between dominant differential bacterial genera and both abnormal behaviors and intestinal TRP metabolites (**Figure 6**). Although impaired amino acid transport at the BBB in NDDs (64, 65) might be expected to reduce CNS access of tryptophan (TRP) and kynurenine (KYN), elevated peripheral levels of these metabolites may facilitate their central delivery via alternative or concentration-dependent mechanisms. This concept is supported by studies demonstrating that increased peripheral KYN, can elevate hippocampal kynurenic acid levels and impair cognitive function (66). In our study, the absence of LAT1 expression changes suggests that such non-canonical pathways may account for the central actions of peripherally derived TRP/KYN in our model. Thus, heightened peripheral KYN/TRP availability could still contribute to NDD-associated neuropathology and behavioral abnormalities (66). Circulating TRP availability is largely determined by the balance between TRP metabolism and absorption in the intestine, modulated by both microbiota and host immunity (67). Our data strongly suggest that the neurobehavioral changes and microglia-activated neuroinflammation induced by prenatal Poly I:C exposure might be associated with the promotion of TRP-KYN metabolism mediated by intestinal microecology. This is further supported by the observed dominance of specific bacteria linked to tryptophan metabolism, such as the increased abundance of Candidatus *Saccharimonas* and the reduced level of *Ruminococcus gauvreauii_group* in Poly I:C-exposed offspring—both of which have been previously implicated in the regulation of tryptophan metabolism (68–70).

Consistent with prior studies reporting anxiety-like behaviors predominantly in male offspring following prenatal Poly I:C exposure (41, 71), our study demonstrates that female offspring also exhibit significant anxiety-like behavior, social deficits, and spatial learning and memory impairments (**Figure 1**). These findings, which are in line with both existing literature (72) and our own previous reports (36, 37), are supported by multiple behavioral tests including the open field, elevated plus maze, and Morris water maze. Microglia, as innate immune effector cells in the brain, are involved in neuroinflammation and contribute to neurodevelopmental disturbances in NDDs, affecting neuronal connectivity and brain homeostasis (73, 74). They mediate the fetal brain’s response to maternal inflammation, playing a crucial and lasting role (75). A previous study by Anand et al. suggested that microglia in the mouse brain express higher levels of Ido1 compared to astrocytes and neurons, playing an important role in protection against toxoplasmic encephalitis and responding to other cytokines (11, 76). In this study, we evaluated the effects of prenatal Poly I:C exposure on microglial immunoreactivity in juvenile female offspring rats by measuring the density, morphology, and specific markers of microglia in the PFC (**Figure 2**). The PFC has been widely accepted to be a brain region implicated in social, cognitive, and affective functions commonly disrupted in NDD patients (77). Similar to other studies, we found no significant change in microglial (Iba1-positive) density, but there was a significant increase in the soma area of Iba1-positive cells in the Poly I:C group (**Figures 2B, C**) and upregulation of genes encoding cytokines associated with pro-inflammatory microglial activity, including *IL-1β*, *Tnf-α*, and *Cd86* (**Figures 2D-F**). These results provide novel evidence that prenatal Poly I:C-induced MIA may cause pro-inflammatory microglial activation in the PFC of female adolescent offspring, which could be highly relevant to their behavioral alterations and neuroimmune disturbance. This aligns with previous observations (78) and supports the presence of central inflammatory processes and microglial anomalies identified in post-mortem brains from subsets of patients with schizophrenia and ASD (79–82). Emerging evidence indicates that MIA-induced inflammation upregulates Ido1, which diverts TRP metabolism toward the KYN pathway. Concurrently, MIA enhances Kmo expression, disrupting the QA/KYNA balance and altering brain TRP-KYN pathway homeostasis (83).

Consistent with most clinical studies on blood from ASD or SZ patients (7, 84–86), and with observations in mouse brains (86), we found elevated KYN concentrations and KYN/TRP ratios in the plasma of Poly I:C rats (**Figure 3**), suggesting increased activity of Ido1 and the KYN pathway induced by prenatal Poly I:C treatment. These results imply that KYN may increase traffic from the periphery into the brain in this model. Since all TRP and approximately 60% of KYN in the brain are transported from the periphery by Lat1 (87, 88), while the remaining 40% of KYN is synthesized from TRP by Ido1 in astrocytes and microglia (89, 90). In this study, the lack of significant difference in Ido1 and Lat1 expression between Poly I:C and Saline rats in the PFC (**Figure 2K**) may suggest that prenatal Poly I:C exposure increases brain KYN due to peripheral KYN pathway activation. Elevated KYN may decrease endogenous TRP degradation via serotonergic-melatonergic pathways, subsequently reducing the availability of metabolites that are closely associated with the physiology and pharmacology of behaviors. Furthermore, our study observed an increase in Kmo in the brain’s PFC (**Figure 2K**), indicating that KYN may be further metabolized into the neurotoxic Kmo branch, producing QA. Our findings of behavioral alterations, microglia-associated neuroinflammation, and KYN pathway activation in both the brain and periphery suggest that Poly I:C-induced pathological abnormalities in MIA offspring may be linked to activated TRP-KYN metabolism.

Since TRP availability largely depends on the balance between catabolism and exogenous absorption in the intestine (32, 91), we evaluated the fecal metabolome by LC-MS. OPLS-DA showed a separation between groups (**Figure 4C**), indicating that the composition of gut metabolites in female offspring was disturbed by prenatal Poly I:C exposure. Enrichment analysis of the differential metabolites (**Figure 4F**) revealed that TRP metabolism was one of the most significantly enriched pathways, demonstrating that prenatal Poly I:C exposure affects the TRP metabolism in the intestinal microenvironment. Interestingly, our fecal metabolomics analysis revealed a notable increase in QA levels in the control group compared to the Poly I:C exposed offspring. The differential changes in QA levels and KMO expression between the intestine and PFC likely result from multifactorial regulation of the kynurenine pathway across tissues. This compartmentalized response may involve tissue-specific substrate availability, enzymatic activity, and inflammatory milieu, ultimately reflecting the complex metabolic adaptation to immune activation along the gut-brain axis. Future studies will specifically explore the mechanisms underlying this compartmentalized response, including tissue-specific substrate availability, enzymatic activity, and inflammatory signaling along the gut-brain axis.

There is increasing recognition of the regulatory roles that dense and diverse microbial communities in the intestine play in TRP metabolism under both physiological and disease conditions (92). This is because the microbiota can directly and indirectly modulate host endogenous TRP metabolism, and variations in TRP metabolism can negatively influence microbial proliferation and microbiota diversity (67, 93). The balance between bacterial TRP metabolism and host TRP absorption determines circulating TRP availability. To further investigate whether gut microbiota modulates intestinal TRP metabolism, we compared gut bacterial composition and diversity by 16S rRNA sequencing. Our findings, consistent with clinical observations in ASD and ADHD patients (94, 95), revealed significant intestinal dysbiosis (**Figure 5**), as indicated by a lower GMHI index Poly I:C offspring (**Figure 5C**).

Although the specific strains of bacteria associated with TRP metabolism remain unclear, recent non-targeted fecal metabolomics analyses suggest that several bacterial genera are linked to TRP availability and pathogenic roles in various diseases. For example, *Enterococcus* is associated with L-TRP production in the intestine (96), Muribaculum is linked to TRP metabolite levels in the brain and is established as a key promoter of 5-HT synthesis (97). Gut-derived lactic acid enhances TRP to 5-HT in regulation of anxiety via *Akkermansia muciniphila*. He et al. found that acetic acid can alleviate metabolic dysfunction caused by sleep disorders; the relative abundance of acetogenic bacteria, such as *Prevotellaceae* and *Dubosiella*, was significantly increased (98). *Ruminococcus* regulates TRP-derived 5-HT (68) and contributes to the pathogenic role of irritable bowel syndrome (99). Candidatus *Saccharimonas* is associated with abnormal TRP metabolism (69, 70) and it is characteristic of the autism model constructed by VPA exposure (100, 101). *Barnesiella* is involved in inflammation in diabetic kidney disease by regulating TRP metabolism (102). Consistent with these reports, our study found correlations between TRP metabolites and bacteria such as *Ruminococcus gauvreauii*_group, Candidatus *Saccharimonas*, norank_f_Flavobacteriaceae, and *Enterococcus* (**Figures 5D, E**). Flavobacteriaceae belongs to the Bacteroidota phylum and has garnered extensive attention in environmental microbiology and aquatic pathology. Although no studies have yet established a direct association between Flavobacteriaceae and NDDs, our study suggests that norank_f_Flavobacteriaceae may play a role in TRP metabolism. This is worthy of further exploration in future research. Importantly, some bacterial genera associated with TRP metabolism, in particular both Candidatus *Saccharimonas* and *Ruminococcus gauvreauii*_grou*p*, were found to be significantly related to the percentage of distance in the target quadrant during the training period of the MWM test, a key parameter of learning and memory evaluation, similar to the observation by Deng et al., who reported that long-term chronic stress can disrupt KYN metabolism along the gut-brain axis, and homeostasis of some microbiota was disrupted (103). In summary, the significant correlation of the *Ruminococcus gauvreauii*_group with improved cognitive performance suggests it may act as a beneficial regulator. Conversely, the strong association of Candidatus *Saccharimonas* with metabolic abnormality and cognitive impairment identifies it as a potential novel inducer of tryptophan metabolic imbalance. These correlations highlight the critical role of the gut microbiota in mediating Poly I:C-induced neuroendocrine immune disturbances.

Prenatal exposure to Poly I:C in resulted in dysregulation of the TRP-KYN pathway and disruption of gut microbiota in offspring. Correlations were observed among microbial alterations, shifts in TRP metabolism, and behavioral deficits. Although MIA is known to directly disrupt tryptophan metabolism at the placental level (104), our study also provides evidence that MIA-induced long-term pathological and neurobehavioral abnormalities in offspring may be attributed to the shift of TRP metabolism toward the KYN pathway, driven by elevated proinflammatory cytokines. Furthermore, our findings suggest that the gut microbiome may serve as an additional and potentially amplifying factor in this dysregulation. The concurrent presence of microbial and metabolic disturbances indicates a possible interaction between these systems. Future studies involving fecal microbiota transplantation or antibiotic interventions will be necessary to establish causal relationships.

Several limitations in this study should be noted. Firstly, this study focused only on female rats, and further studies in males are critical to understanding potential gender differences. Secondly, considering the effects of behavioral experiments on rat physiology, we collected feces before the behavioral experiments and blood at the time of euthanasia. It is unknown whether these results from different time points are consistent, and further study is required. Thirdly, due to the limited volume of plasma and PFC tissue samples collected from the offspring, we were unable to perform additional analyses. Future studies incorporating targeted metabolomics to quantify QA and KYNA levels in both plasma and specific brain regions will be crucial to conclusively define the neurotoxic shift following MIA. Fourth, it should be noted that all rats were purchased as pregnant dams. Despite a standard one-week acclimatization period to minimize transportation stress, this duration may have been insufficient for their gut microbiota to fully stabilize in the new environment. This incomplete adaptation represents a potential source of variability. Future studies employing in-house breeding or extended acclimatization periods would help to clarify these effects. Finally, we acknowledge that the application of the GMHI, which was developed based on human metagenomic data, to a rat model represents a limitation of our study. While the index provided valuable insights in our experimental context, there are inherent differences between human and rodent gut microbiomes in terms of composition and functional capacity. Thus, the interpretation of GMHI values in rodent studies should be made with appropriate caution. Future studies aimed at developing and validating species-specific microbial indices would be beneficial for preclinical research.

## 5 Conclusion

Our findings provide evidence that microglia activation is associated with behavioral changes and prolonged neuroinflammation induced by prenatal Poly I:C exposure in juvenile female rats. Importantly, these neuropathologic and behavioral characteristics were found to be accompanied by enhanced TRP-KYN metabolism, along with a disturbance of intestinal microecology. These results support the role of the microbiota-gut-brain axis in neurodevelopmental disorders and strongly suggest that the tryptophan pathway could be a key molecular mechanism.

## Data Availability

The datasets presented in this study can be found in online repositories. The names of the repository/repositories and accession number(s) can be found in the article/**Supplementary Material**.
