# Supplementary material for: Prenatal Poly I:C exposure affects tryptophan-kynurenine metabolism associated with intestinal microbiome in female juvenile rats
Source: Front Immunol. 2025 Oct 1;16:1669845. doi: 10.3389/fimmu.2025.1669845 (PMC12521143; doi:10.3389/fimmu.2025.1669845)
Supplement: Supplementary file 1 [file Table1.docx]

Supplementary Material

# Supplementary Data

**a. Timeline of animal treatment**

**
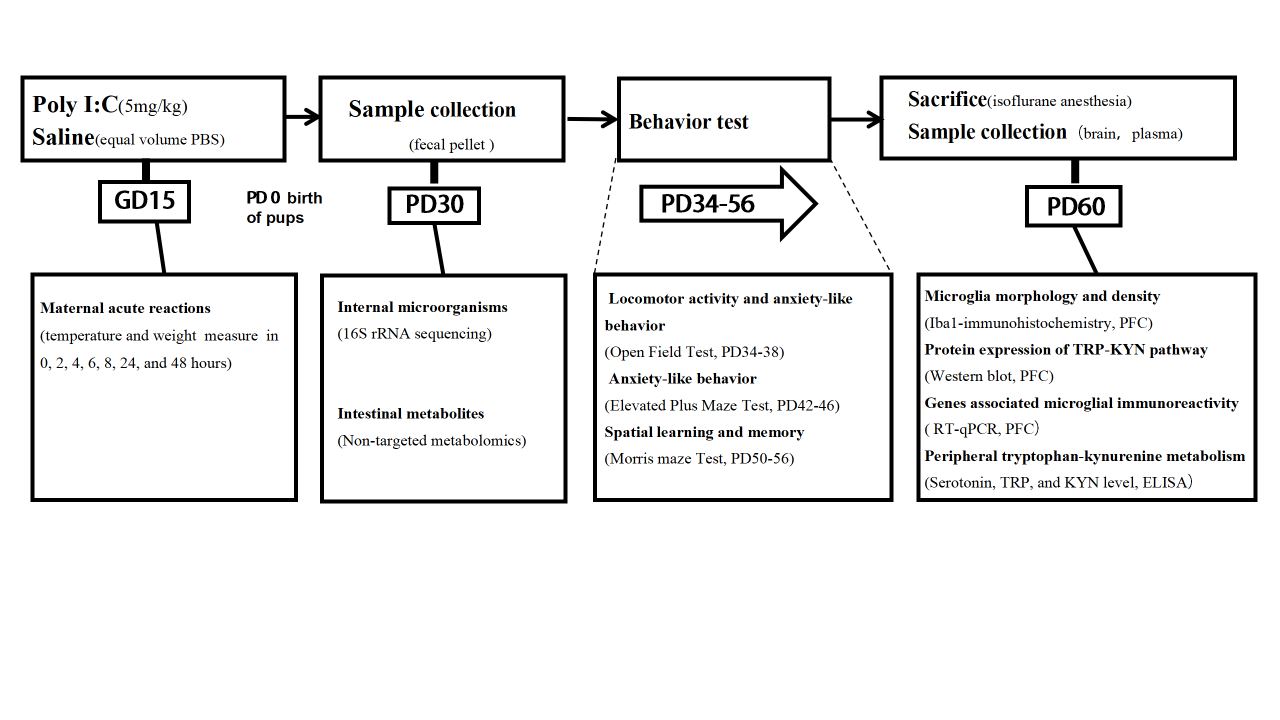
**

Note: Poly I:C, Polyinosinic acid; GD, Gestational day; PD, Postnatal day; PFC, Prefrontal cortex; TRP, Tryptophan; KYN, Kynurenine; ELISA, Enzyme-linked immunosorbent assay;RT-qPCR, Reverse transcription-quantitative polymerase chain reaction.

**b.Animals detail for experiments**

| **Group** | **Dam** | **Anal temperature/**  **Body weight** | **Female offspring** | **Feces^a^** | **Behavior**  **test** | **Brain^b^** | **Blood^c^** | **Brain^d^** |
| --- | --- | --- | --- | --- | --- | --- | --- | --- |
| Poly I:C | 933 | √ | 322 | √ | √ |  | √ |  |
|  |  |  | 323 |  | √ |  |  | √ |
|  | 937 | √ | 321 |  | √ |  |  |  |
|  |  |  | 334 | √ | √ |  | √ | √ |
|  | 939 | √ | 325 | √ | √ |  | √ |  |
|  |  |  | 326 |  | √ |  |  | √ |
|  | 940 | √ | 327 | √ | √ |  | √ | √ |
|  |  |  | 328 |  | √ | √ |  |  |
|  | 941 | √ | 329 | √ | √ | √ | √* |  |
|  |  |  | 330 |  | √ |  |  |  |
|  | 942 | √ | 332 |  | √ | √ |  |  |
|  |  |  | 333 | √ | √ |  | √ |  |
|  | 943 | √ | 324 |  | √ |  |  | √ |
|  |  |  | 331 | √ | √ | √ | √ |  |
|  | 944 | √ | 335 |  | √ |  |  |  |
|  |  |  | 336 | √ | √ |  | √ | √ |
| Saline | 503 | √ | 341 | √ | √ |  | √ |  |
|  |  |  | 343 |  | √ |  |  | √ |
|  | 586 | √ | 342 | √ | √ |  | √ | √ |
|  |  |  | 346 |  | √ | √ |  |  |
|  | 587 | √ | 344 |  | √ |  |  | √ |
|  |  |  | 345 | √ | √ | √ | √* |  |
|  | 588 | √ | 347 |  | √ |  |  | √ |
|  |  |  | 348 | √ | √ |  | √ |  |
|  | 589 | √ | 350 |  | √ | √ |  |  |
|  |  |  | 351 | √ | √ |  | √ | √ |
|  | 590 | √ | 349 |  | √ |  |  | √ |
|  |  |  | 353 | √ | √ |  | √ |  |
|  | 591 | √ | 354 |  | √ | √ |  |  |
|  |  |  | 355 | √ | √ |  | √ |  |
|  | 592 | √ | 352 (dead) |  |  |  |  |  |
|  |  |  | 356 | √ | √ |  | √ |  |

Note: Poly I:C, Polyinosinic acid; a, 16S rRNA/LC-MS, 16S rRNA and Liquid Chromatography-Mass Spectrometry; b, IHC, Immunohistochemistry; c, ELISA, Enzyme-linked immunosorbent assay; d, RT-qPCR/WB, Reverse transcription-quantitative polymerase chain reaction and Western Blot;

* severe hemolysis sample discarded for ELISA analysis.

**Supplementary Figure S1** Experimental design. a.Timeline of animal treatment, b. Details of animal assignment in each experiment. Sixteen pregnant Sprague-Dawley rats were randomly divided into two groups for single intraperitoneal injection of Poly l:C (5 mg/kg, n = 8) or saline (equal amount of PBS, Control; n = 8) at GD15. To reduce the abortion risk induced by blood collection, none invasive processes including anal temperature and body weight changes at 0, 2, 4, 6, 8, 24, and 48h of post-injection were applied for monitoring Poly I:C-induced acute reaction in pregnant rats. Half of the female offspring were used for this study (Poly I:C: 16, Saline: 16). During PD34-58, all animals performed behavioral tests, including open field (PD34-36), elevated plus maze (PD51-52), and Morris maze (PD55-58). Prior to this, 8 animals from each group were randomly selected to collect stool for both intestinal microorganisms and metabolites analysises by 16S rRNA sequencing and non-targeted metabolomics (LC-MS) at PD30, respectively. At PD60, all animals were euthanized by isoflurane anesthesia, brain tissues were collected for microglial immunoreactivity analysis by combining Iba1-immunohistochemistry (n = 4) and RT-qPCR/WB (n = 6), and plasma was collected for peripheral metabolites of tryptophan metabolism measuring by ELISA (n = 8). To minimize the litter effects, the animals selected for the same type of sample collection were from different dams.

**Supplementary Figure S2** OTU level of Alpha Diversity index including A. Ace, B. Shannon, C. Chao, and D. Simpson; rarefaction curves of E. Sobs and F. Shannon index. Based on the OTU information, alpha diversity indices including Ace, Shannon, Chao, and Simpson index, rarefaction curves of Sobs, and Shannon index were calculated with Mothur v1.30.2. Statistical T-test was used to compute whether there was a significant difference in the index values between the two groups. A certain number of sequences were randomly selected from the samples, and the Alpha diversity index corresponding to these sequences was calculated, the rarefaction curves were drawn with the amount of data extracted as the horizontal coordinate and the Alpha diversity index value as the vertical coordinate.


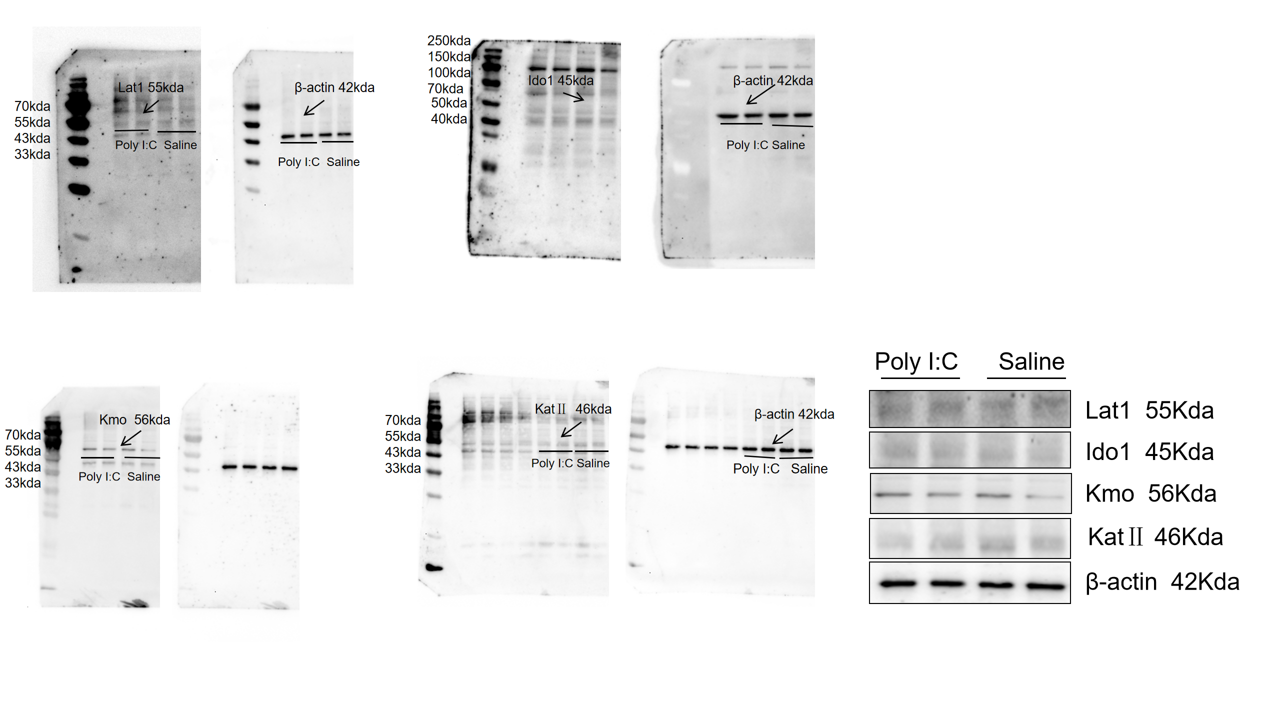
**Supplementary Figure S3** Original Western blot images

**Supplementary Table S1** Primer sequences for gene expression in RT-qPCR

| Genes | Forward primer | Reverse primer |
| --- | --- | --- |
| *Tnf-α* | GGCTTTCGGAACTCACTGGA | GGGAACAGTCTGGGAAGCTC |
| *Cd86* | AGACATGTGTAACCTGCACCAT | ACTTTTTCCGGTCCTGCCAA |
| *Il-1β* | TCCTCTGTGACTCGTGGGAT | TCAGACAGCACGAGGCATTT |
| *Cd206* | TGATTCCGGTCGCTGTTCAA | GAACGGAGATGGCGCTTAGA |
| *TGF-β2* | CGAAACTGTCTGCCCAGTTG | TGTAGAAAGTGGGCGGGATG |
| *TGF-β3* | GCCTCAGTCTTTGGGATCTGG | ATGTGTGAACCCAGGAACGA |
| *β-actin* | CCCGCGAGTACAACCTTCTT | CGCAGCGATATCGTCATCCA |
| *Gadph* | GCATCTTCTTGTGCAGTGCC | TACGGCCAAATCCGTTCACA |

**Supplementary Table S2** Results of the behavior tests index

| **Behavior test** | **Behavior test index** | **Poly I:C group** | **Saline group** | ***P*-value** |
| --- | --- | --- | --- | --- |
| Open Field test | rearing frequency | 21.380 ± 1.963 | 25.690 ± 1.957 | 0.142 |
|  | total moving distance in the arena(cm) | 4670 ± 291.900 | 4047 ± 244.800 | 0.125 |
|  | total moving distance in the central zone(cm) | 429.600 ± 42.920 | 432.300 ± 74.610 | 0.976 |
|  | accumulated moving time in the arena(s) | 337.600 ± 19.690 | 317.600 ± 13.600 | 0.269 |
|  | accumulated time spent in the central zone(s) | 31.880 ± 3.951 | 43.940 ± 7.113 | 0.160 |
|  | frequency of center zone entry | 23.000 ± 2.724 | 22.690 ± 3.896 | 0.949 |

**Supplementary Table S3** Gene expression of anti-inflammatory（M2）related markers of microglia in PFC between the rats in the group of Poly I:C and Saline.

| **Anti-inflammatory marker** | **Poly I:C group** | **Saline group** | ***P*-value** |
| --- | --- | --- | --- |
| *Tgf-β2* | 1.095 ± 0.105 | 0.998 ± 0.207 | 0.258 |
| *Tgf-β3* | 1.505 ± 0.140 | 1.002 ± 0.269 | 0.061 |
| *Cd206* | 1.748 ± 0.262 | 1.003 ± 0.228 | 0.057 |

**Supplementary Table S4** The optimized sequences information and OTU identified in this study during gut 16s RNA sequencing.

| **Group** | **ID** | **Seq_num** | **Base_num** | **Mean_length** | **OTU_num** |
| --- | --- | --- | --- | --- | --- |
| Poly I:C | 322 | 60379 | 25560153 | 423.329 | 314 |
|  | 325 | 61120 | 25716176 | 420.749 | 380 |
|  | 327 | 59508 | 25329863 | 425.655 | 363 |
|  | 329 | 51105 | 21629493 | 423.236 | 349 |
|  | 331 | 59240 | 25354972 | 428.004 | 199 |
|  | 333 | 66692 | 28401249 | 425.857 | 305 |
|  | 334 | 51534 | 21947500 | 425.884 | 267 |
|  | 336 | 62150 | 26355415 | 424.061 | 379 |
| Saline | 341 | 65329 | 27135638 | 415.369 | 268 |
|  | 342 | 78844 | 32842672 | 416.553 | 491 |
|  | 345 | 63184 | 26632373 | 421.505 | 247 |
|  | 348 | 62622 | 26557114 | 424.086 | 330 |
|  | 351 | 59390 | 25192615 | 424.190 | 337 |
|  | 353 | 53994 | 22903202 | 424.181 | 394 |
|  | 355 | 64877 | 26999071 | 416.158 | 269 |
|  | 356 | 63697 | 26781164 | 420.446 | 330 |
| Total |  | 983665 | 415338670 |  | 5222 |
| Mean ± SE |  | 61479 ± 1635 |  | 422.500 ± 0.934 |  |

Note： Raw FASTQ files of sixteen randomly selected fecal samples from the two groups (n = 8) were obtained by 16s RNA sequencing. The optimized sequences information, including total numbers, total length, and mean length, were de-multiplexed from raw FASTQ files using an in-house Perl script, and then quality-filtered by fastp version 0.19.6 and merged by FLASH version 1.2.11. Then the optimized sequences with 97% sequence similarity level were clustered as an operational taxonomic unit (OTU) using UPARSE 11.
